# Statistical methods for exploring spontaneous adverse event reporting databases for drug-host factor interactions

**DOI:** 10.1186/s12874-023-01885-w

**Published:** 2023-03-27

**Authors:** Zhiyuan Lu, Ayako Suzuki, Dong Wang

**Affiliations:** 1 Division of Bioinformatics and Biostatistics, National Center for Toxicological Research, U.S. Food and Drug Administration, Jefferson, Arkansas USA; 2 Division of Gastroenterology, Duke University, Durham, North Carolina USA; 3 Department of Medicine, Durham VA Medical Center, Durham, North Carolina USA

**Keywords:** Drug-host factor interactions, Likelihood ratio tests, FAERS, Postmarket surveillance, Spontaneous reporting adverse event databases

## Abstract

**Background:**

Drug toxicity does not affect patients equally; the toxicity may only exert in patients who possess certain attributes of susceptibility to specific drug properties (i.e., drug-host interaction). This concept is crucial for personalized drug safety but remains under-studied, primarily due to methodological challenges and limited data availability. By monitoring a large volume of adverse event reports in the postmarket stage, spontaneous adverse event reporting systems provide an unparalleled resource of information for adverse events and could be utilized to explore risk disparities of specific adverse events by age, sex, and other host factors. However, well-formulated statistical methods to formally address such risk disparities are currently lacking.

**Methods:**

In this paper, we present a statistical framework to explore spontaneous adverse event reporting databases for drug-host interactions and detect risk disparities in adverse drug events by various host factors, adapting methods for safety signal detection. We proposed four different methods, including likelihood ratio test, normal approximation test, and two tests using subgroup ratios. We applied our proposed methods to simulated data and Food and Drug Administration (FDA) Adverse Event Reporting Systems (FAERS) and explored sex-/age-disparities in reported liver events associated with specific drug classes.

**Results:**

The simulation result demonstrates that two tests (likelihood ratio, normal approximation) can detect disparities in adverse drug events associated with host factors while controlling the family wise error rate. Application to real data on drug liver toxicity shows that the proposed method can be used to detect drugs with unusually high level of disparity regarding a host factor (sex or age) for liver toxicity or to determine whether an adverse event demonstrates a significant unbalance regarding the host factor relative to other events for the drug.

**Conclusion:**

Though spontaneous adverse event reporting databases require careful data processing and inference, the sheer size of the databases with diverse data from different countries provides unique resources for exploring various questions for drug safety that are otherwise impossible to address. Our proposed methods can be used to facilitate future investigation on drug-host interactions in drug toxicity using a large number of reported adverse events.

**Supplementary Information:**

The online version contains supplementary material available at 10.1186/s12874-023-01885-w.

## Background

Drug toxicity does not affect patients equally; the toxicity may only exert in patients who possess certain attributes of susceptibility to specific drug properties (i.e., drug-host interaction). This concept is crucial for drug safety but has been under-studied, primarily due to methodological challenges and the difficulty to obtain data. Moreover, understanding the effect of host factors is important for identifying the mechanism of the adverse reaction and planning future drug development. For example, there is great interest in clarifying the role that sex and age play in drug induced liver toxicity for various drugs. The spontaneous adverse event reporting databases could be utilized to explore risk disparities of specific adverse events by age, sex, and other host factors. However, well-formulated statistical methods specifically to address such risk disparities are currently lacking as the need is different from safety signal detection, which has received significant attention regarding spontaneous reporting databases for postmarket surveillance.

Spontaneous adverse event reporting databases have played a critical role in detecting safety signals for postmarket surveillance. As the number of patients meeting screening criteria in clinical trials is always limited and the follow up time is necessarily short, some adverse effects can only be discovered by monitoring the relevant reports after the drug has been marketed to the larger population. Spontaneous adverse event (AE) reporting databases continuously collect reports of adverse events and can provide real time surveillance. Important examples include the Food and Drug Administration (FDA) Adverse Event Reporting Systems (FAERS); the VigiBase, an international database from the World Health Organization; the Vaccine Adverse Event Reporting System (VAERS) by FDA and the Centers of Disease Control and Prevention (CDC). There has long been interest in mining spontaneous reporting databases for safety signals by detecting drug-AE combinations with unusually high report counts. Due to the unique challenges for analyzing this type of data, a significant literature has been developed on statistical methods for this task. Examples include proportional reporting ratios [1], reporting odds ratios [2], the likelihood ratio tests [3–8], and Bayesian methods [9–12]. Most of these methods produce a score for each drug-AE combination and compare this score to a critical value (threshold). A score exceeding the critical value indicates disproportionally high reporting rate for a drug-AE combination, which can indicate an association between the drug and AE that can be further investigated with other approaches. The null hypothesis here is that there is no association between the drug and AE. One popular method to determine the critical value is based on the contingency table as in Table 1. The baseline frequency

$$\begin{aligned} E_{ij}=\frac{n_{i\cdot }n_{\cdot j}}{n_{\cdot \cdot }} \end{aligned}$$

is defined as the expected value of the report count $n_{ij}$, for AE *i* and drug *j*, under the null hypothesis of no association between the drug and AE. Assuming that the null distribution for $n_{ij}$ is $\text {Poisson}(E_{ij})$, $n_{ij}$ values much larger than $E_{ij}$ as predicted by the Poisson distribution indicate evidence for drug-AE association. If multiple hypotheses are to be tested, ideally, the critical value should be chosen to control family-wise error rate (FWER) or false discovery rate (FDR, in the presence of multiple comparisons). However, this can be difficult to achieve for some of the methods (see [3] for more discussion).

**Table 1:** The $2\times 2$ table for the event reporting counts of drug *j* and AE *i*

	Drug *j*	No Drug *j*	Total
AE *i*	$n_{ij}$	$n_{i\cdot }-n_{ij}$	$n_{i\cdot }$
No AE *i*	$n_{\cdot j}-n_{ij}$	$n_{\cdot \cdot }-n_{i\cdot }-n_{\cdot j}+n_{ij}$	$n_{\cdot \cdot }-n_{i\cdot }$
Total	$n_{\cdot j}$	$n_{\cdot \cdot }-n_{\cdot j}$	$n_{..}$

One aspect of analyzing spontaneous reporting data is regarding the adjustment for covariates. With the main goal of detecting safety signals in the overall population, several authors proposed approaches to adjust for covariates in constructing the tests. These include stratification-based approaches where baseline frequencies for each drug-AE combination was adjusted by averaging over stratum-specific baseline frequencies [3, 10, 11], using logistic regression to adjust for covariates [6, 13], and an approach based on propensity score matching [14]. In a related line of research, Sandberg and coworkers [15]explored using subgroup disproportionality analysis to detect safety signal in subgroups defined by covariates (also see [16]).

The question that we want to address in this paper is different from those considered in aforementioned references. Specifically, our emphasis is not on safety signal detection, rather, we focus on investigating drug-host factor interactions regarding specific AEs. The null hypothesis is that * the occurrence of an AE is independent of the status of the covariate (e.g., sex or age) of interest*. Thus, the effect of the covariate is central to the investigation rather than to be adjusted away (i.e., effect modification). Though current approaches such as subgroup disproportionality analysis [15] do provide hints at the association between host factors and AE, the focus is still signal detection and the association is not explicitly tested with proper controls for Type I error. Researchers have found creative ways to obtain information for this type of questions (e.g., the ANOVA tests [17], the subgroup disproportionality analysis [15]), these methods were not specifically designed to test this hypothesis and thus do not have the relevant significance levels. For this purpose, we are especially interested in methods that properly control FWER or FDR.

Though we use the FAERS database in our analysis, the general principle will apply to other postmarket surveillance databases as well. FAERS is a database that contains spontaneous adverse event reports that are submitted to FDA, it supports the FDA’s post marketing safety surveillance program for drug and therapeutic biologic products. In this paper, we demonstrate the possibility of utilizing FAERS data to explore the effect of host factors and drug properties on the risk of adverse reactions, which can improve the understanding of adverse effects of drugs on the market as well as inform future drug development practices. The disparities for sex and age regarding liver toxicity is used as an example in this paper. Drug liver toxicity can result in serious clinical outcomes including acute liver failure and the need for liver transplantation. It is a common cause for drug withdrawals from the market. Identifying patterns in drug liver toxicity and individuals at risk for liver damage remains challenging despite tremendous research effort. There is a long running interest in host factors that may modify an individual’s risk for liver toxicity and might be involved in drug-host interactions [18, 19].

The paper is structured as following. We first describe contingency table based methods for modeling drug-host factor interactions in the setting of spontaneous reporting databases and inference procedures are proposed. Some simulation results are then given to evaluate the proposed methods. Analysis using actual data from FAERS is described to demonstrate the proposed method in real applications. We conclude with some general discussion.

## Methods

Just like in the signal detection case, we use the $2\times 2$ table as the starting point to construct hypothesis tests. Suppose we are interested in the disparity in reported AE counts regarding different levels of a host factor. Common examples for the host factor include sex (male or female), age (above 65 years or below 65 years), and other variables for demographic or health status. For simplicity, we assume that the host factor has two levels, though extensions can be made to multi-level cases, especially for the likelihood ratio test (see Additional file 1 for an example). There are potentially two ways to construct the $2\times 2$ table in this setting. In one approach, regarding a specific AE of interest, we use the contingency table to test for drugs with unusual disparities regarding the host factor (Table 2). In another approach using Table 3, on the other hand, we might consider a specific drug and test for AEs that demonstrate unusual host factor disparity. Both approaches might be of interest depending on the study. For a concrete example, we consider liver toxicity as the AE of interest. We can test for drugs with significant sex or age disparities (Table 2), or we can try to identify drugs for which the liver toxicity demonstrates unusual patterns regarding the host factor in comparison with other AEs (Table 3). The two approaches have different properties, which we will discuss with real data analysis. Next, we will present mathematical details for the hypothesis test using Table 2. For tests based on Table 3, the test statistic will be readily available by straightforward modifications.

**Table 2:** The $2\times 2$ table for the event counts of drug *j* and a host factor given a specific AE *i*, where (1) denotes Group 1 and (2) denotes Group 2 based on a host factor such as sex or age

	Group 1	Group 2	Total
drug *j*	$n^{(1)}_{ij}$	$n^{(2)}_{ij}$	$n_{ij}$
no drug *j*	$n^{(1)}_{i\cdot }-n^{(1)}_{ij}$	$n^{(2)}_{i\cdot }-n^{(2)}_{ij}$	$n_{i\cdot }-n_{ij}$
Total	$n^{(1)}_{i\cdot }$	$n^{(2)}_{i\cdot }$	$n_{i\cdot }$

**Table 3:** The $2\times 2$ table for the event reporting counts of AE *i* and a host factor given a specific drug *j*, where (1) denotes Group 1 and (2) denotes Group 2

	Group 1	Group 2	Total
AE *i*	$n^{(1)}_{ij}$	$n^{(2)}_{ij}$	$n_{ij}$
no AE *i*	$n^{(1)}_{\cdot j}-n^{(1)}_{ij}$	$n^{(2)}_{\cdot j}-n^{(2)}_{ij}$	$n_{\cdot j}-n_{ij}$
Total	$n^{(1)}_{\cdot j}$	$n^{(2)}_{\cdot j}$	$n_{\cdot j}$

Consider a given AE (say *i*) and drug *j*. We denote $n^{(s)}_{ij}$ to be the number of reports for the *i*th AE and the *j*th drug regarding patients of the host factor group *s* ($s=1$ for Group 1 and $s=2$ for Group 2). For sex, the group is male or female. For age, it could be above or below 65 years. Now consider the *J* $2\times 2$ tables indexed by drug (with drug *j* or without drug *j*) and the host factor group (1 or 2) as in Table 2. We assume that $n^{(s)}_{ij}$ follows a $\text {Poisson}(\mu ^{(s)}_{ij})$ distribution with $\mu ^{(s)}_{ij}=\lambda ^{(s)}_{ij}E^{(s)}_{ij}$. Here, the baseline frequency $E^{(s)}_{ij}={n^{(s)}_{i\cdot }n_{ij}}/{n_{i\cdot }}$ is analogous to that used by other authors [3, 10], it is the expected count for each group if there is no association between the drug and the host factor (when the null hypothesis is true). When the host factor has no effect, one would expect that $\lambda ^{(1)}_{ij}=\lambda ^{(2)}_{ij}=1$ for all *j*, while $\lambda ^{(1)}_{ij}\ne \lambda ^{(2)}_{ij}$ for some *j* is indicative of drug-host factor interactions. We want to identify significant patterns of disparity in reporting frequencies between host factor levels. The global hypothesis is thus $H_{0}: \lambda ^{(1)}_{ij}=\lambda ^{(2)}_{ij}=1$ for all *j*, versus $H_{a}: \lambda ^{(1)}_{ij}\ne \lambda ^{(2)}_{ij}$ for at least one *j*. Under the null hypothesis, the sum of independent Poisson random variables, $n_{ij}=n^{(1)}_{ij}+n^{(2)}_{ij}$, is also a Poisson random variable. Hence, conditioning on row and column totals, $n^{(1)}_{ij}$ has a binomial distribution with parameters $n_{ij}$ and probability ${n^{(1)}_{i\cdot }}/n_{i\cdot }$; that is,

1 $$\begin{aligned} n^{(1)}_{ij}|n_{ij},n^{(1)}_{i\cdot }, n_{i\cdot } \thicksim \text {Binomial}\Big (n_{ij},\frac{n^{(1)}_{i\cdot }}{n_{i\cdot }}\Big ), \end{aligned}$$

and $$n^{(2)}_{ij}=n_{ij}-n^{(1)}_{ij}.$$

This framework allows us to perform inference on spontaneous reporting data with host factors. There are potentially multiple ways to construct statistical tests regarding the null hypothesis. We will consider several different test statistics and evaluate their performances in the simulation studies.

### Likelihood ratio test

Under the null hypothesis, $\lambda ^{(s)}=1$ for $s=1,2$. Under $H_{a}: \lambda ^{(1)}_{ij}\ne \lambda ^{(2)}_{ij}$ for at least one *j*, as the maximum likelihood estimator (MLE) for $\mu ^{s}_{ij}$ is $n^{s}_{ij}$, and the MLE for $\lambda ^{(s)}_{ij}$ is thus $n^{(s)}_{ij}/E^{(s)}_{ij}$. Under $H_{0}: \lambda ^{(1)}_{ij}=\lambda ^{(2)}_{ij}=1$ for all *j*, the MLE for $\mu ^{(s)}_{ij}$ is $E^{(s)}_{ij}$. Based on the Poisson distribution, the likelihood fuction is

$$\begin{aligned} L(\lambda )\propto \prod _{s} \big \{\mu ^{(s)}\big \}^{n^{(s)}}. \end{aligned}$$

Correspondingly, we can derive the log likelihood ratio statistic as

$$\begin{aligned} LR_{ij}= & {} -\log \left( \frac{\max _{H_{0}}L(\lambda )}{\max _{H_{a}}L(\lambda )}\right) \\= & {} -n_{ij}\log \left( \frac{n_{ij}}{n_{i\cdot }}\right) +n^{(1)}_{ij}\log \left( \frac{n^{(1)}_{ij}}{n^{(1)}_{i\cdot }}\right) +n^{(2)}_{ij}\log \left( \frac{n^{(2)}_{ij}}{n^{(2)}_{i\cdot }}\right) . \end{aligned}$$

To test for the global null hypothesis, the maximal value of $LR_{ij}$ among all drugs can be computed as

$$\begin{aligned} MLR_i=\max _{j}(LR_{ij}), \end{aligned}$$

which is also useful for the control of FWER procedure described in the Multiple inference section. When the likelihood ratio statistic exceeds certain critical value based on the null distribution, it indicates a discrepancy from the null hypothesis. The distribution of the various $LR_{ij}$ statistics and/or the $MLR_i$ variable would be needed to calculate *p*-values as well as determine the critical value. As their distributions are not readily available in an analytic form, we utilize Monte Carlo simulations, similar to that in [3], to approximate them. Specifically, for any given AE-drug combination *i*, *j*, we can generate simulated data sets and obtain values $LR_{ij,1},LR_{ij,2},\dots ,LR_{ij,m}$ using the distribution given in (1), where *m* is a large number. We can then calculate the *p*-value of any value $\ell r$ for each drug as

2 $$\begin{aligned} \frac{1}{m}\sum _{k=1}^{m}\mathbbm {1}\left( \ell r\ge LR_{ij,k} \right) , \end{aligned}$$

where $\mathbbm {1}(\cdot )$ is the indicator function. Similarly, Monte Carlo simulation can be carried out similar to that in [3] to obtain the null distribution for $MLR_{i}$ for the control of family wise error rate, which is further discussed in subsequent sessions.

### Normal approximation test for the group proportions

As described in Equation (1), for every AE *i* and drug *j*, conditioning on the totals, we have $n_{ij}^{(1)}$ following independent binomial distributions with parameters $n_{ij}$ and $n^{(1)}_{\cdot j}/n_{\cdot j}$. We apply a commonly used tool for constructing statistical test, that is, as $n_{ij}$ becomes large, the distributions of the z-scores (difference from mean of a statistic as measured in its standard deviation) approaches the standard normal distribution (normal distribution with mean zero and standard deviation 1) under the null distribution. Recall that under the null hypothesis, the host factor has no effect on the reporting frequencies. As the mean and the standard deviation is available from the binomial distribution, we have

3 $$\begin{aligned} z_{ij}:=\sqrt{n_{ij}}\left( \frac{n_{ij}^{(1)}}{n_{ij}}-\frac{ n_{i\cdot }^{(1)} }{n_{i\cdot }} \right) \Bigg / \sqrt{ \frac{ n_{i\cdot }^{(1)} }{n_{i\cdot }}\left( 1-\frac{ n_{i\cdot }^{(1)} }{n_{i\cdot }}\right) }\overset{approx.}{\sim } N(0,1). \end{aligned}$$

From these approximations, one can obtain the *p*-value for each individual two sided test, that is, $\hat{p}_{ ij }:=P\{ |X|>|z_{ij}|\}$ for $X\sim N(0,1)$, and multiple hypothesis testing can be performed using these values as discussed subsequently. In contrast with the likelihood ratio test, no Monte Carlo simulation will be needed when using the normal approximation. Another way to view this test is to treat it as testing for the difference in proportions, we provide details in Additional file 2.

### Subgroup ratios

The reporting odds ratio (ROR) and the proportional reporting ratio (PRR) analyses are two established approaches for safety signal detection using $2\times 2$ count tables [1, 2] as in Table 1. PRR is defined as risk ratio between two groups to quantify the strength of association between a drug and an event and ROR is defined as odds ratio between two groups to quantify the strength of association [3, 5]. These approaches can easily be extended to the detection of disparities regarding host factors in adverse event databases.

For each fixed AE *i* and drug *j* in Table 2, the counts are tabulated by the levels regarding the drug (drug or no drug), and the two different subpopulation groups determined by the host factor. The PRR would therefore take the form of

$$\begin{aligned} PRR_{ij}:=\frac{n^{(1)}_{ij}/n_{ij}}{(n^{(1)}_{i\cdot }-n^{(1)}_{ij})/(n_{i\cdot }-n_{ij})} \end{aligned}$$

while the ROR would take the form of

$$\begin{aligned} ROR_{ij}:=\frac{n^{(1)}_{ij}/n^{(2)}_{ij}}{(n^{(1)}_{i\cdot }-n^{(1)}_{ij})/(n^{(2)}_{i\cdot }-n^{(2)}_{ij})}. \end{aligned}$$

To approximate their null hypothesis distributions, we observe that, for large values of $n_{ij}$ under the null hypothesis, the approximation in (3) simplifies to

$$\begin{aligned} \sqrt{n_{ij}}\left( \frac{n^{(1)}_{ij}}{n_{ij}} -\frac{n_{i\cdot }^{(1)}}{n_{i\cdot }} \right) \overset{approx.}{\thicksim } N(0,1). \end{aligned}$$

Using this approximation, we can use the Delta method [20] to obtain the following normal approximations for the PRR:

$$\begin{aligned} \sqrt{n_{ij}}\left[ \log PRR_{ij}- \log \left( \frac{p_{i}}{ \left( n_{i\cdot }^{(1)}-n_{ij}p_{i}\right) /\left( n_{i\cdot }-n_{i\cdot }^{(1)} \right) } \right) \right] \overset{approx.}{\thicksim } N(0,\sigma _{PRR,ij}^2), \end{aligned}$$

where

$$\begin{aligned} \sigma _{PRR,ij}^2:=p_{i}(1-p_{i})\left( \frac{ n_{i\cdot }^{(1)} }{ p_{i}\left( n_{i\cdot }^{(1)}-n_{ij}p_{i} \right) }\right) ^2 \text { and } p_{i}:=\frac{ n^{(1)}_{i\cdot } }{ n_{i\cdot } }. \end{aligned}$$

More details are provided in Additional file 2.

Similarly, for large $n_{ij}$, the ROR have the approximate distribution

$$\begin{aligned}{} & {} \sqrt{n_{ij}}\left[ \log ROR_{ij}- \log \left( \frac{p_{i}/(1-p_{i})}{ \left( \frac{n_{i\cdot }^{(1)}}{n_{ij}}-p_{i} \right) /\left( \frac{n_{i\cdot }^{(2)}}{n_{ij}}-(1-p_{i}) \right) } \right) \right] \\\overset{approx.}{\thicksim } & {} N(0,\sigma _{ROR,ij}^2), \end{aligned}$$

where

$$\begin{aligned} \sigma _{ROR,ij}^2:=p_{i}(1-p_{i})\left[ \frac{n^{(1)}_{i\cdot }/n_{ij}}{p_{i}\left( \frac{n^{(1)}_{i\cdot }}{n_{ij}}-p_{i} \right) } +\frac{n^{(2)}_{i\cdot }/n_{ij}}{(1-p_{i})\left( \frac{n^{(1)}_{ij}}{n_{ij}}-(1-p_{i}) \right) } \right] ^2. \end{aligned}$$

Using these approximations, we can perform the corresponding tests for any drug and AE regarding the host factor using straight computations based on the normal distribution.

### Multiple inference

We have outlined several possible test statistics along with their approximate distributions. For the multiple inference problem in this setting (i.e., testing involving multiple drugs or AEs), Huang et al. (2011) [3] and others [4, 7, 8] have focused on controlling FWER by rejecting the null hypothesis based on the maximal statistic. This is a step-down procedure belonging to the class of max-t tests [21]. To summarize this procedure, given non-negative valued test statistics $T_1,T_2,...,T_J$ and an $\alpha \in (0,1)$, let $T_{max}:=\max _{j=1,...,J} T_j$ and let $T_{max,\alpha }$ be the $1-\alpha$ quantile of $T_{max}$ under the global null hypothesis (under which every $T_j$ follows the null distribution). With these notations, the global null hypothesis is rejected if $T_{max}>T_{max,\alpha }$, this is followed by the rejection of individual null hypotheses $H_{0,j}$ for every *j* where $T_{j}>T_{max,\alpha }$. We will implement this method here for all our outlined test statistics and refer to it as the Max-Stat method. For the LR values, the quantiles of the maximal statistic, $MLR_{i}$, can be obtained by Monte Carlo simulations with a procedure similar to [3]. For other test statistics, the quantile can be computed using the distribution function of the standard normal distribution. Here, we need the $1-\alpha$ quantile of the maximum of the absolute values of *J* independent standard normal random variables, which can be approximated by the $(1-\alpha/2 )^{1/J}$ quantile of the standard normal distribution.

An alternative approach for multiple testing would be to control the false discovery rate (FDR). This can be performed using the Benjamini-Hochberg [22] method, where the *p*-value of the *J* tests are arranged in increasing order as $P_{(1)}\le P_{(2)}\le ...\le P_{(J)}$ and the overall null hypothesis is rejected if there are any $1\le \ell \le J$ such that $P_{(\ell )}\le \frac{\ell }{J}\alpha$. If there is a rejection, one would reject all null hypothesis associated with $P_{(1)},P_{(2)},...,P_{(k)}$, where *k* is the maximum integer satisfying $P_{(k)}\le \frac{k}{J}\alpha$. We implement this approach by computing the *p*-values for the likelihood ratio test through Monte Carlo simulation for each drug, and the *p*-values for the other outlined methods are computed with the normal approximations. We refer to it as the BH method in subsequent text.

### FAERS dataset and preprocessing

We focus on the FAERS dataset to perform real data analysis for evaluating the methods outlined in the previous subsections as well as to anchor our simulation studies. Each year, over one million adverse events associated with the use of drug or biological products are entered into the database. It thus constitutes a rich source of information for the risk of adverse events for drugs and biological products on the market. The FAERS dataset is publicly available as a quarterly download on the FDA website (https://www.fda.gov/drugs/questions-and-answers-fdas-adverse-event-reporting-system-faers/fda-adverse-event-reporting-system-faers-latest-quarterly-data-files). For each event report, besides information for the drug and adverse event, FAERS also contain demographic information for the patient as well as that of therapy and patient outcomes. The data is contained in seven tables regarding information for the patient, drugs, adverse events, and other information concerning reports submitted to the FAERS system. The FAERS data are complex and requires extensive preprocessing. We used a procedure similar to [23] to process the data consisted of reports from January 2004 to June 2015. This resulted in 4,928,413 unique cases.

The reports were first grouped by the AEs (defined as MedDRA (Medical Dictionary for Regulatory Activities) terms). Within each group of reports of AE *i*, we tabulated the number of reports of drug *j* versus no drug *j* for each drug, as outlined in Table 2. The groups that we use, denoted as (1) and (2) for the columns of the same table, are regarding sex (male versus female) or age (under 65 years old versus at least 65 years old). To generate these tables, only drugs listed as the primary suspect in a report are counted. For either analysis, the missing data entries (missing sex or age entries, respectively) were removed and the counts were tabulated. Furthermore, we filtered out the AE-drug combinations (*i*, *j*) for which the expected counts are low. We performed this step because in some of our exploratory analysis, we found out that including all such entries resulted in extremely inaccurate results when performing inference. Hence, we left out the drug-AE combinations where any of the 4 cells in the $2 \times 2$ table is less than 5. After this step, there remains a large number of AEs attributed to a small number of drugs, i.e., for many AE *i*, $n_{ij}\ne 0$ for only a small set of drugs *j*. As we want to evaluate the ability of the proposed methods to identify true disparities among a large number of null hypotheses, we focus on AEs that have more than 5 associated drugs and used them as the base for simulation studies. For subsequent simulation studies, we also left out a small number of AEs *i* for which there exists a drug *j* where $n_{ij}>\min \{n^{(1)}_{i\cdot }, n^{(2)}_{i\cdot }\}$. As mentioned in the subsection for LRT, this situation might result in very rare cases of drawing $n_{ij}^{(s)}$ values larger than $n_{i\cdot }^{(s)}$, which will in turn cause an error in calculating subgroup ratios. This problem is only limited to the simulation study as our inference on subgroup ratios is based on the normal approximation in data analysis. Similar processing was also carried out for the real data examples regarding liver toxicity.

### Simulation setup

There are many adjustable parameters for the simulation, namely the total of AE reports, the number of reports for each drug, and the total number of reports for each group regarding the host factor. To obtain values for these parameters, we utilized the actual counts obtained from the processed FAERS data as described in the Methods section. This will allow the simulation to reflect real world scenarios.

We performed simulations using counts taken from individual AEs as the simulation parameters regarding both sex and age. The processed FAERS dataset contains 2033 unique AEs that are tabulated with drugs and sex. Instead of simulating the counts for every AE, which would be computationally intensive, we chose a subset of AEs and performed simulation based on their contingency tables.

First, we divided the AEs into three categories depending on the size of the total report counts. The distribution of $n_{i\cdot }$ among the AEs has $25\%$ and $50\%$ quantiles of 674 and 1337 respectively, so we denote AEs with $n_{i\cdot }$ within the intervals (0, 674], (674, 1337], and $(1337,\infty )$ to be “small”, “moderate”, and “large” count sizes, respectively. We use this setup because the test’s behavior should be very homogeneous once there the number of AEs is large and we want to emphasize the lower part of the spectrum for the number of AEs. Within each count size group, we also applied different simulation settings in terms of deviations from the null hypothesis: with the null hypothesis value set at $p_{i}=n_{i\cdot }^{(1)}/n_{i\cdot }$, the corresponding value under the alternative hypothesis is set to be

4 $$\begin{aligned} p_{ij}'=p_{i}+\Delta \cdot (-1)^{\mathbbm {1}(p_{i}> 0.5)}, \end{aligned}$$

with $\mathbbm {1}(\cdot )$ being the indicator function, and where $\Delta$ can be taken to be 0, 0.05, 0.1, or 0.2. Here, a larger $\Delta$ value means a larger deviation from the null hypotheses. The values are chosen so that we can cover a range of deviations from being negligible to being significant but still realistic.

For each of the 3 categories of $n_{i\cdot }$, we randomly selected 250 AEs (denoted $i_1,\dots ,i_{250}$). For each selected AE $i_k$, the associated drugs (corresponding to the primary suspect in real data analysis) from the preprocessed FAERS dataset were identified, denoted as $j_1,\dots ,j_{J_{k}}$. We randomly selected 1/5 of these drugs (rounded to the nearest whole number) to follow the alternative hypothesis proportion given in (4), and the remaining drugs were assumed to follow the null hypothesis. Specifically, for $k=1,\dots ,250$ we randomly selected drugs $j_{\ell _1},\dots ,j_{\ell _{m_k}}$ from $\{ j_1,\dots ,j_{J_k} \}$, where $m_k$ equals $J_k/5$ rounded to the nearest integer, and simulate

5 $$\begin{aligned} n_{i_k j}^{(1)}\sim Binomial\left( n_{i_k j},p_{i_k j}' \right) \qquad&\text {if }j\in \{ j_{\ell _1},\dots ,j_{\ell _{m_k}} \}\nonumber \\ n_{i_k j}^{(1)}\sim Binomial\left( n_{i_k j},p_{i_k } \right) \qquad&\text {if }j\notin \{ j_{\ell _1},\dots ,j_{\ell _{m_k}} \}, \end{aligned}$$

where $p_{i}$ and $p_{ij}'$ are defined as in (4). Under this scheme, the AE-drug-sex (or AE-drug-age) counts were simulated 500,000 times, with 2000 iterations allocated to each of the 250 AEs. Each of the inference methods described in Methods was applied on every simulation iteration.

### Method for the analysis of liver toxicity data

As liver toxicity is a complex phenomenon with a number of manifestations, we created the liver toxicity event by combining groups of 53 “Preferred Terms” (codes from MedDRA), which are listed in Additional file 1. More information for how the liver toxicity term is defined can be found in references [17, 24]. We applied the proposed tests to the processed data for liver toxicity, with the male and female sex defining the two different subpopulations. As the effect of sex on liver toxicity is still not well understood, the analysis here is only for demonstrating how to use the proposed method. A thorough dissection of the sex (or age) disparity patterns for liver toxicity will require more studies using multiple experimental and clinical approaches.

We first calculated the empirical FWER for simulation under the global null hypothesis. This is calculated as the number of simulation runs with one or more rejected null hypotheses divided by the total number of simulation runs, $M=500,000$. Under the alternative hypotheses $(\Delta >0)$, we used false discovery rate and sensitivity to measure the performance of the four different methods, likelihood ratio test (LRT), normal approximation, proportional reporting ratio (PRR), and reporting odds ratio (ROR). False discovery rate (FDR) is the average proportion of falsely detected signals out of all detected signals, which can be estimated as the following for *M* simulations:

$$\begin{aligned} \sum\limits^{M}_{l=1}\frac{\#\text { of falsely rejected null hypotheses in simulation }l}{\#\text { of rejected null hypotheses in simulation }l}/M, \end{aligned}$$

where the quantity inside the summation sign is set to zero if no null hypothesis is rejected for that simulation run. Sensitivity is the average proportion of the number of correctly rejected null hypotheses among all true alternative hypotheses, estimated as

$$\begin{aligned} \sum\limits^{M}_{l=1}\frac{\#\text {of correctly rejected null hypotheses in simulation}l}{\#\text {of all true alternative hypotheses in simulation}l}/M. \end{aligned}$$

## Results

### Simulation studies

We focus our presentation to the simulation results regarding sex. The results regarding age give essentially the same conclusions, which we present in Additional file 1. Table 4 reports the empirical FWER for the four different tests using either Max-Stat or BH adjustment for multiple testing when all null hypotheses are true ($\Delta =0$). It shows that both LRT and the normal approximation methods performed very well. Both PRR and ROR demonstrated much higher empirical FWER than the nominal values across sample sizes, which is consistent with observations in the literature for safety signal detection. The results are similar for BH or Max-Stat adjustment, which is not surprising as FWER is equivalent to FDR when all null hypotheses are true [22]. As all null hypotheses are true, sensitivity is not available in this simulation setting.

**Table 4:** Empirical family-wise error rate (FWER) for the likelihood ratio test (LRT), normal approximation, proportional reporting ratio (PRR), and reporting odds ratio while using either Max-Stat or Benjamini-Hochberg (BH) method for adjustment of multiple testing when all null hypotheses are true. The inference methods were applied at $\alpha =0.05$. The result is based on 500,000 simulation runs based on the FAERS dataset regarding sex for different sample sizes when there is no difference between host factor groups ($\Delta =0$)

Method	$\boldsymbol{n}_{\boldsymbol{i}\cdot}$ size	LRT	Normal approx.	PRR	ROR
BH	small	0.0363	0.0421	0.136	0.107
	medium	0.0376	0.0416	0.160	0.127
	large	0.0491	0.0388	0.341	0.271
Max-Stat	small	0.0465	0.0416	0.136	0.106
	medium	0.0479	0.0412	0.159	0.126
	large	0.0492	0.0385	0.335	0.266

Tables 5 and 6 report the sensitivity and FDR for the four methods for $\Delta =0.025, 0.05, 0.10,$ or 0.20 for three different $n_{i\cdot }$ groups when using BH (Table 5) or Max-Stat (Table 6) adjustment for multiple testing. Some general patterns can be observed from the tables. Across groups for different $n_{i\cdot }$ sizes, the sensitivity generally rises when the $\Delta$ values grow. With BH adjustment, the values of FDR stay slightly below 0.05 for LRT and normal approximation method, while PRR and POR both resulted in inflated FDR. Not surprisingly, using the Max-Stat adjustment sometimes yielded smaller FDR values than using the BH adjustment as the former controls for the more stringent family wise error rate criterion. This is most prominent for large $n_{i\cdot }$ and $\Delta$ values. On the other hand, the sensitivity tends to be lower when using Max-Stat adjustment for large values of $\Delta$ and $n_{i\cdot }$.

**Table 5:** Sensitivity and false discovery rate (FDR) for the likelihood ratio test (LRT), normal approximation, proportional reporting ratio (PRR), and reporting odds ratio methods while using the Benjamini-Hochberg (BH) method for adjustment of multiple testing under different parameter settings. The simulation takes counts from 250 random AEs from each $n_i.$ size category and randomly assigns $20\%$ of drugs from each AE to follow a proportion that differs from the null hypothesis proportion by a value of $\Delta$

	$\boldsymbol{n}_{\boldsymbol{i}\cdot}$ size	$\Delta$	LRT	Normal approx.	PRR	ROR
Sensitivity	small	0.025	0.00821	0.0106	0.0297	0.0185
	small	0.05	0.0146	0.0192	0.0449	0.0260
	small	0.1	0.0501	0.0625	0.108	0.0700
	small	0.2	0.244	0.278	0.351	0.305
	medium	0.025	0.00598	0.00831	0.0238	0.0125
	medium	0.05	0.0139	0.0194	0.0436	0.0199
	medium	0.1	0.0621	0.0784	0.130	0.0718
	medium	0.2	0.298	0.340	0.435	0.336
	large	0.025	0.0031	0.00417	0.0156	0.00645
	large	0.05	0.0143	0.0180	0.0401	0.0169
	large	0.1	0.0759	0.0899	0.150	0.0828
	large	0.2	0.315	0.356	0.465	0.358
FDR	small	0.025	0.0299	0.0345	0.110	0.0877
	small	0.05	0.0299	0.0345	0.110	0.0882
	small	0.1	0.0298	0.0348	0.106	0.0866
	small	0.2	0.0303	0.0356	0.0978	0.0822
	medium	0.025	0.0303	0.0338	0.129	0.103
	medium	0.05	0.0302	0.0338	0.127	0.102
	medium	0.1	0.0302	0.0342	0.121	0.0999
	medium	0.2	0.0299	0.0348	0.103	0.0875
	large	0.025	0.0383	0.0308	0.263	0.215
	large	0.05	0.0357	0.0308	0.241	0.203
	large	0.1	0.0317	0.0316	0.187	0.160
	large	0.2	0.0304	0.0338	0.125	0.100

**Table 6:** Sensitivity and false discovery rate (FDR) for the likelihood ratio test (LRT), normal approximation, proportional reporting ratio (PRR), and reporting odds ratio methods while using the Max-Stat method for adjustment of multiple testing under different parameter settings. The simulation takes counts from 250 random AEs from each $n_i.$ size category and randomly assigns $20\%$ of drugs from each AE to follow a proportion that differs from the null hypothesis proportion by a value of $\Delta$

	${\boldsymbol n}_{\boldsymbol i\cdot}$ size	$\Delta$	LRT	Normal approx.	PRR	ROR
sensitivity	small	0.025	0.00925	0.00999	0.0279	0.0168
	small	0.05	0.0158	0.0183	0.0424	0.0237
	small	0.1	0.0523	0.0600	0.103	0.0652
	small	0.2	0.250	0.268	0.338	0.290
	medium	0.025	0.00684	0.00783	0.0217	0.0112
	medium	0.05	0.0152	0.0184	0.0401	0.0176
	medium	0.1	0.0643	0.0740	0.120	0.0645
	medium	0.2	0.293	0.311	0.402	0.301
	large	0.025	0.00311	0.0039	0.0132	0.00555
	large	0.05	0.0133	0.0162	0.0328	0.0142
	large	0.1	0.0644	0.0726	0.115	0.0650
	large	0.2	0.240	0.260	0.369	0.257
FDR	small	0.0250	0.0382	0.0340	0.110	0.0863
	small	0.05	0.0380	0.0338	0.109	0.0865
	small	0.1	0.0370	0.0330	0.104	0.0838
	small	0.2	0.0329	0.0287	0.0911	0.0723
	medium	0.025	0.0385	0.0333	0.128	0.102
	medium	0.05	0.0381	0.0329	0.125	0.101
	medium	0.1	0.0366	0.0311	0.116	0.0967
	medium	0.2	0.0281	0.0235	0.0871	0.0732
	large	0.025	0.0389	0.0299	0.256	0.211
	large	0.05	0.0368	0.0277	0.230	0.198
	large	0.1	0.0290	0.0213	0.162	0.148
	large	0.2	0.0152	0.0111	0.087	0.0679

In regard to the performance of individual methods, the LRT and normal approximation methods display similar results in terms of sensitivity and false discovery rate for every parameter configuration. The ROR and the PRR inference methods have similar or slightly higher (for PRR) sensitivity in simulations under alternative hypotheses, but with substantially increased FDR in some cases, sometimes up to twice of its nominal value.

### Real data analysis

In this section, we apply the proposed inference methods to the actual values of report counts regarding liver toxicity in FAERS. First, we applied Table 2 based tests on a group of analgesics to identify drugs with significant disparities regarding sex. Analgesics are widely used for treatment of symptoms of pain and inflammation, ranging from the common cold to osteoarthritis and rheumatoid arthritis. Though liver toxicity events caused by analgesics are very rare, it is an important concern due to the shear amount of medicine used [25]. Here, we apply tests based on Table 2 on report counts for analgesics in the FAERS database. Of analgesics with reports in FAERS, 12 of them (acetaminophen, aspirin, ibuprofen, meloxicam, etodolac, indomethacin, ketoprofen, diclofenac, ketorolac, piroxicam, nabumetone, and naproxen) passed the preprocessing criteria described in the FAERS dataset and preprocessing section. The results for likelihood ratio test and normal approximation tests using Max-Stat adjustment are shown in Table 7. Of the 12 drugs included in the analysis, four of them (acetaminophen, aspirin, ibuprofen, and meloxicam) have adjusted *p*-values below 0.05 by the Max-Stat method with acetaminophen being the most significant. Results for all four tests described in the Methods section are provided in Additional file 3.

**Table 7:** Likelihood ratio test and normal approximation test results for analgesics. The columns are drug name, report count for male without the drug (No Drug/Male), report count for female without the drug (No Drug/Female), report count for male with the drug (Drug/Male), report count for female with the drug (Drug/Female), the likelihood ratio statistic with the adjusted *p*-value (in parentheses), and the z-score for the normal approximation test with the corresponding *p*-value. The Max-Stat method was used to adjust the *p*-values for multiple testing

Drug name	No Drug Male	No Drug Female	Drug Male	Drug Female	LRT(adj.p)	Normal Approx.(adj.p)
Acetaminophen	46798	51289	1032	1789	67.2388(0.0000)	11.5059(0.0000)
Aspirin	47695	52984	135	94	6.1345(0.0062)	3.5011(0.0055)
Ibuprofen	47566	52706	264	372	4.4538(0.0409)	2.9749(0.0346)
meloxicam	47818	53044	12	34	4.3995(0.0433)	2.8949(0.0446)
Etodolac	47821	53053	9	25	3.1308(0.1452)	2.4440(0.1610)
Indomethacin	47820	53053	10	25	2.5873(0.2604)	2.2308(0.2683)
Ketoprofen	47821	53073	9	5	0.8066(0.9482)	1.2654(0.9370)
Diclofenac	47625	52822	205	256	0.7963(0.9504)	1.2604(0.9387)
Ketorolac	47824	53067	6	11	0.5090(0.9867)	0.9996(0.9898)
Piroxicam	47821	53063	9	15	0.4782(0.9925)	0.9713(0.9920)
nabumetone	47825	53069	5	9	0.3902(0.9975)	0.8756(0.9968)
Naproxen	47750	52982	80	96	0.1337(1.0000)	0.5168(1.0000)

We also performed Table 3 based tests for liver toxicity regarding drugs in the FAERS database. After the preprocessing step, 596 drugs meet the criteria described previously. In Table 8, we list the 24 drugs found to be significant using the likelihood ratio test $(\alpha =0.05)$ with Max-Stat adjustment for multiple testing. As before, the result will be very similar if the normal approximation test is used. In addition to the 24 drugs in Table 8, the normal approximation test will also flag venlafaxine and sildenafil to be significant. Drugs deemed significant by using each one of the four tests are listed in tables given in Additional file 4.

**Table 8:** Drugs with significant sex disparities for liver toxicity as identified by the likelihood ratio test based on Table 3. The Max-Stat method is used for adjustment of multiple testing $(\alpha =0.05)$. The columns are drug name, report count for male without liver toxicity (No liver tox./Male) for each drug, report count for female without liver toxicity (No liver tox./Female) for each drug, report count for male with liver toxicity (Liver tox./Male), report count for female with liver toxicity (Liver tox./Female), and the likelihood ratio statistic (LR)

Drug name	No liver tox. Male	No liver tox. Female	Liver tox. Male	Liver tox. Female	LRT
Leuprolide	4405	5341	133	24	51.35646
Isotretinoin	6044	7457	423	253	39.15879
Etanercept	43723	124146	614	1168	30.12383
Rosuvastatin	8153	11178	565	498	23.20041
Fingolimod	2330	9148	172	338	22.62352
Azithromycin	1653	2633	147	101	19.28138
Esomeprazole	7125	16566	180	234	15.94716
Adalimumab	42601	96825	658	1135	15.02019
Cyclosporine	4716	5530	489	385	14.63681
Amlodipine	4776	7825	156	134	14.32332
Metformin	4896	6604	390	346	14.20642
Sorafenib	5250	2100	1107	299	14.00521
Aripiprazole	5592	7428	134	91	12.07196
Doxorubicin	1349	2671	120	125	10.91474
Propranolol	659	1113	48	26	10.64977
Amoxicillin-clavulanate	1287	1708	417	367	10.31652
Insulin Glargine	6233	8495	87	55	10.0872
Clarithromycin	1751	2674	190	174	10.06727
Doxycycline	770	1446	92	84	9.622339
Valsartan	3883	6148	155	146	9.48397
Ranitidine	1750	2945	75	58	9.404383
Topotecan	364	717	42	27	9.303118
Fluvastatin	348	305	80	151	8.956207
Peginterferon Alfa-2b	4750	4086	286	163	8.23285

## Discussion and conclusions

It is challenging to identify and study drug adverse events that are relatively rare. As there is a limit on the sample size for clinical trials, many AEs are not discovered until after the drug has been approved through postmarket monitoring. As a result, spontaneous reporting databases like FAERS are extremely useful for studying drug safety due to their large report counts and comprehensiveness. The limitations [5] of FAERS and other spontaneous reporting databases have also been noted, which include over-reporting, under-reporting, incomplete information, replicated information, other potential biases, and no guarantees of causal relationship. Beside using preprocessing steps to improve the quality of information, it is commonly acknowledged that findings of FAERS data analysis have to be further analyzed before being accepted for use in decision making [3, 4]. Despite these difficulties, the postmarket surveillance database has proved to be immensely valuable for drug safety studies [14, 26].

Identifying host factor-drug interactions is an important topic for drug safety. Clarifying the effects of host factors like sex and age on drug AEs can not only contribute to the scientific understanding of toxicity mechanisms but also aid safety considerations in future drug development. Though some questions in this area could be answered with animal models and in vitro studies, the availability of human data is of great importance to answer questions with human relevance. For this purpose, postmarket surveillance databases also have great potential, though they have not been adequately utilized in practice. Similar to the application in safety signal detection, data must be processed with care, and results need to be compared with other sources of information. Unlike safety signal detection, the hypothesis of interest for investigations of host factor-drug interaction is different and requires a different statistical framework, which we addressed in this paper. We expect that FAERS and other databases to serve as valuable resources for hypothesis generation and corroboration in the study of host factor-drug interactions for drug safety due to the huge number of AE reports.

Similar to the case for safety signal detection, one challenge in studying host factor-drug interaction using spontaneous reporting data is that FAERS or other databases cannot provide information on the total number of prescriptions for each drug to patients defined by the host factor group. Like many methods used for safety signal detection, we circumvent this problem by constructing a baseline under the null hypothesis using $2\times 2$ contingency tables. The difference with the case for safety signal detection is that now we have two different ways of constructing the contingency table: by drug or by AE, corresponding to Tables 2 and 3 respectively. As discussed earlier, tests based on Table 2 will compare drugs for a specific AE, but is vulnerable to the bias caused by unbalanced prescription patterns. For example, an analysis regarding sex disparities using Table 2 based tests for all drugs in FAERS tends to flag drugs for breast cancer and prostate cancer treatment. However, if limited to a class of drugs with similar prescription patterns, these tests are useful to identify drugs with unusual disparities. Tests based on Table 3 are less prone to the bias caused by prescription patterns. But as it compares the AE of interest to other AEs, it might not detect the disparity patterns if the disparity is shared across AEs for the drug. Table 3 based tests might also flag some AEs that predominantly appear in male or females. In this case, the relative strength of disparity across drugs is more useful for hypothesis generation. One should consider the application context to ameliorate potential confounding. Just as in safety signal detection, these tests are most useful when used in combination with other sources of information for the drug.

For tests based on contingency tables, we proposed the likelihood ratio test, normal approximation test, and two tests based on subgroup ratios. In addition, we applied the Max-Stat method and the Benjamini-Hochberg method for the adjustment of multiple testing. In our simulation study, the first two tests give similar results and satisfactory control of FDR with sensitivity increasing with the size of the parameter $\Delta$ under the alternative hypothesis. This is expected since, by definition, a larger $\Delta$ value means a larger deviation from the null hypotheses. Given the large number of reports in spontaneous reporting databases, normal approximation should be sufficient for these tests in most applications. The two methods based on subgroup ratios, PRR and ROR, have much higher FDR than other methods. This is consistent with observations in safety signal detection. As expected, Max-Stat adjustment is more conservative than the BH method. Users can make choices between the two according to their needs. We have provided code for important functions in our computation in Additional file 5.

The results show that one can obtain similar performance using either the normal approximation or the likelihood ratio tests, but using the likelihood ratios require some special attention. First, there is the extra effort of performing Monte Carlo simulations to approximate the distributions of the likelihood ratios, while using a Gaussian distribution only requires standard calculations. Secondly, the simulated distribution of the likelihood ratios is discrete in contrast to the continuous Gaussian distributions. Extra care needs to be taken to when calculating the *p*-value as in expression (2), and using the “>” instead of “$\ge$” has, in our experience, resulted in inflated FDRs when using the BH adjustment. It should also be noted that when $n_{ij} > n_{i\cdot }^{(s)}$ for one of the group *s*, while not affecting the computation of the likelihood ratio, it is possible to draw $n_{ij}^{(s)}$ values larger than $n_{i\cdot }^{(s)}$ in the Monte Carlo simulation. This is rare and only makes the *p*-value slightly more conservative when it happens. But it does mean that one should use care when the host groups are highly unbalanced and one drug accounts for a major portion of reports of a specific AE. On the other hand, the likelihood ratio test provides a natural path for extension to more complicated tests such as the case for host factors with multiple levels (see Additional file 1).

In this paper, we presented analysis for liver toxicity as an example due to its importance in drug development. Sex disparity for adverse effects is of great interest for researchers, we focused on it as the host factor for simulation and real data analysis. In real data analysis, we applied Table 2 based test on analgesics. Consistent with our findings, it has been reported that women are more likely than men to have acetaminophen-induced liver injury and the potential mechanism has been discussed in the literature (e.g., [27, 28]). Evidence also exists for aspirin, ibuprofen, and meloxicam as well [29–31]. The results for LRT and normal approximation test are very similar, which is consistent with the findings in simulation studies. We also applied Table 3 based tests to identify drugs for which liver toxicity demonstrates significant unbalance regarding sex relative to other AEs. These drugs (shown in Table 8) include drugs with well documented sex disparities for liver toxicity (etanercept, fingolimod, amoxicillin-clavulanate), drugs with known sex related differences in drug metabolisms (e.g., cyclosporine, amlodipine, azithromycin), and drugs with known interactions with sex hormones (leuprolide, isotretinoin). There are multiple drugs related to immune response and blood pressure represented in the list.

Simulation based on age as a host factor give very similar results (see Additional file 1). We expect the proposed method (especially the likelihood ratio test) to be applicable to a wide range of AEs and host factors when data are available, though specific biological background for the drug and AE has to be considered in each application. Two especially interesting problems are the effect of race and ethnicity, and the effect of common comorbidities such as diabetes and high blood pressure. These information, however, is not always available in spontaneous reporting systems; but some creative solutions are possible, such as using concomitant drugs to infer comorbidities. The analysis in this paper did not consider age by sex disparities (before and after menopause), so reproductive-state-specific sex differences are not reflected in the results, which we plan to explore in future analysis. A related problem is to test for whether a drug property is associated with the tendency for an AE. For example, one might want to test whether drugs generating reactive metabolites tend to be associated with increased liver toxicity events. We plan to report finding for this area in future communications. Despite their limitations and complexities for analysis, with carefully formulated statistical methods, spontaneous report databases can serve as rich data sources for hypothesis generation and corroboration for a range of problems regarding drug safety.

## Supplementary Information

**Additional file 1.** Appendix with Tables A.1-A.5 and an example for host factors with more than two levels.

**Additional file 2.** Mathematical derivations.

**Additional file 3.** Results for all tests for analgesics.

**Additional file 4.** Results for all tests for liver toxicity.

**Additional file 5.** Code for important functions.

## Data Availability

The FDA FAERS data are publicly available at (https://www.fda.gov/drugs/surveillance/questions-and-answers-fdas-adverse-event-reporting-system-faers), the R code for simulation and analysis is available from D. Wang.
