# Supplementary material for: Statistical methods for exploring spontaneous adverse event reporting databases for drug-host factor interactions
Source: BMC Med Res Methodol. 2023 Mar 27;23:71. doi: 10.1186/s12874-023-01885-w (PMC10041785; doi:10.1186/s12874-023-01885-w)
Supplement: Supplementary file 1 — Additional file 1. Appendix with Tables A.1-A.5 and an example for host factors with more than two levels. [file 12874_2023_1885_MOESM1_ESM.pdf]

Appendix
Statistical methods for exploring spontaneous adverse event reporting databases for drug-host factor interactions

Zhiyuan Lu, Ayako Suzuki, Dong Wang

Appendix

Table A.1: The MedDRA (Medical Dictionary for Regulatory Activities) preferred terms used to define liver toxicity (George et al. 2018; Suzuki et al. 2015). If any report contains one of the terms listed in the table as the adverse event, the patient is considered to have experienced liver toxicity.

Blood alkaline phosphatase increased	Blood bilirubin increased
Transaminases increased	Alanine aminotransferase increased
Aspartate aminotransferase increased	Hepatitis toxic
Hepatitis	Acute hepatic failure
Hepatic failure	Hepatic function abnormal
Cholestatic liver injury	Hyperbilirubinaemia
Jaundice	Liver function test abnormal
Urine bilirubin increased	Hepatitis fulminant
Hepatotoxicity	Hepatocellular injury
Hepatitis acute	Hepatic enzyme increased
Yellow skin	Bilirubin conjugated increased
Cholestasis	Liver injury
Hepatic necrosis	Urobilinogen urine increased
Hepatic enzyme abnormal	Ocular icterus
Hypertransaminasaemia	Jaundice cholestatic
Alanine aminotransferase abnormal	Hepatitis cholestatic
Coma hepatic	Mixed liver injury
Subacute hepatic failure	Bilirubin urine
Blood bilirubin abnormal	Liver transplant
Aspartate aminotransferase abnormal	Drug-induced liver injury
Blood alkaline phosphatase abnormal	Hepatorenal failure
Transaminases abnormal	Reye's syndrome
Hepatorenal syndrome	Hepatic infiltration eosinophilic
Cholestatic pruritus	Jaundice hepatocellular
Total bile acids increased	Glutamate dehydrogenase increased
Icterus index increased	Bilirubin conjugated abnormal
5'nucleotidase increased	

Simulation results for age

In Tables A.2-A.4, we present simulation results when the population is divided by age group (above or up to 50 years old) rather than sex. The simulation settings are very similar to those for sex. One difference is on the cutoff points for sample size categories. After processing and filtering the data to obtain count tables with satisfactory numbers for different age groups, we are left with 739 different AEs. With the values 8874 and 20880 being the 25% and 50% quantiles of the $n_{i.}$'s, we divided the AEs into three categories depending on the size of $n_{i.}$. The $n_{i.}$'s within the intervals $(0, 8874]$, $(8874, 20880]$, and $(20880, \infty)$ are considered to be of "small", "moderate", and "large" count sizes, respectively. Simulations then follow the procedure for those regarding sex. The performance of different inference methods also parallels that observed in simulations regarding sex.

Table A.2: False discovery rate (FDR) for the likelihood ratio test (LRT), normal approximation, proportional reporting ratio (PRR), and reporting odds ratio (ROR) while using either Max-Stat or Benjamini-Hochberg (BH) method for adjustment of multiple testing when all null hypotheses are true. The inference methods were applied at $\alpha = 0.05$. The result is based on 500,000 simulation runs based on the FAERS dataset regarding age for different sample sizes when there is no difference between age groups ($\Delta = 0$).

Method	$n_{i.}$ size	LRT	Normal approx.	PRR	ROR
BH	small	0.0371	0.0445	0.162	0.112
	medium	0.0375	0.0438	0.209	0.141
	large	0.0466	0.0451	0.458	0.303
Max-Stat	small	0.0472	0.0440	0.160	0.110
	medium	0.0480	0.0431	0.206	0.140
	large	0.0492	0.0446	0.451	0.299

Table A.3: Sensitivity and false discovery rate (FDR) for tests regarding disparities for age (defined as above or below 65 years) in the simulation study. The four tests are the likelihood ratio test (LRT), normal approximation, proportional reporting ratio (PRR), and reporting odds ratio methods while using the Benjamini-Hochberg (BH) method for adjustment of multiple testing under different parameter settings. The simulation takes counts from 250 random AEs from each $n_{i.}$ size category and randomly assigns 20% of drugs from each AE to follow a proportion that differs from the null hypothesis proportion by a value of Δ.

	$n_{i.}$ size	Δ	LRT	Normal approx.	PRR	ROR
Sensitivity	small	0.0250	0.00829	0.0119	0.0202	0.0166
	small	0.05	0.0169	0.0250	0.0220	0.0243
	small	0.1	0.0664	0.0879	0.0536	0.0769
	small	0.2	0.297	0.346	0.209	0.324
	medium	0.025	0.00648	0.0102	0.0140	0.0114
	medium	0.05	0.0175	0.0268	0.0160	0.0188
	medium	0.1	0.0838	0.112	0.0553	0.0790
	medium	0.2	0.367	0.427	0.253	0.373
	large	0.025	0.00344	0.00531	0.00773	0.00595
	large	0.05	0.0183	0.0252	0.0146	0.0174
	large	0.1	0.0986	0.124	0.0616	0.0889
	large	0.2	0.396	0.455	0.242	0.394
FDR	small	0.025	0.0301	0.036	0.130	0.0905
	small	0.05	0.0300	0.0361	0.130	0.0901
	small	0.1	0.0302	0.0362	0.128	0.0889
	small	0.2	0.0296	0.0365	0.119	0.0826
	medium	0.025	0.0300	0.0351	0.169	0.114
	medium	0.05	0.0301	0.0353	0.168	0.112
	medium	0.1	0.0299	0.0354	0.163	0.107
	medium	0.2	0.0299	0.0359	0.143	0.0913
	large	0.025	0.0362	0.0367	0.373	0.242
	large	0.05	0.0340	0.0361	0.358	0.225
	large	0.1	0.0317	0.0350	0.300	0.172
	large	0.2	0.0308	0.0359	0.206	0.104

Table A.4: Sensitivity and false discovery rate (FDR) for tests regarding disparities for age (defined as above or below 65 years) in the simulation study. The four tests are the likelihood ratio test (LRT), normal approximation, proportional reporting ratio (PRR), and reporting odds ratio methods while using the Max-Stat method for adjustment of multiple testing under different parameter settings. The simulation takes counts from 250 random AEs from each $n_{i.}$ size category and randomly assigns 20% of drugs from each AE to follow a proportion that differs from the null hypothesis proportion by a value of Δ.

	$n_{i.}$ size	Δ	LRT	Normal approx.	PRR	ROR
Sensitivity	small	0.025	0.00935	0.0113	0.0187	0.0151
	small	0.05	0.0189	0.0238	0.0202	0.0221
	small	0.1	0.0710	0.0845	0.0494	0.0712
	small	0.2	0.303	0.332	0.194	0.305
	medium	0.025	0.00725	0.00964	0.0124	0.0101
	medium	0.05	0.0184	0.0253	0.0139	0.0164
	medium	0.1	0.0846	0.105	0.0480	0.0703
	medium	0.2	0.353	0.389	0.222	0.329
	large	0.025	0.0032	0.00476	0.00621	0.00501
	large	0.05	0.0158	0.0212	0.0111	0.0138
	large	0.1	0.0791	0.0966	0.0464	0.0664
	large	0.2	0.300	0.341	0.173	0.278
FDR	small	0.025	0.0383	0.0356	0.129	0.0893
	small	0.05	0.0381	0.0352	0.129	0.0887
	small	0.1	0.0367	0.0337	0.127	0.0861
	small	0.2	0.0308	0.0279	0.115	0.0724
	medium	0.025	0.0380	0.0344	0.167	0.113
	medium	0.05	0.0378	0.0340	0.166	0.111
	medium	0.1	0.0352	0.0310	0.159	0.104
	medium	0.2	0.0253	0.0220	0.133	0.0756
	large	0.025	0.0387	0.0355	0.368	0.239
	large	0.05	0.0359	0.0320	0.353	0.222
	large	0.1	0.0270	0.0225	0.291	0.162
	large	0.2	0.0132	0.0104	0.184	0.0730

Table A.5: The 2×3 table for the event counts of drug j and a host factor given a specific AE i, where (1) denotes Group 1, (2) denotes Group 2, and (3) denotes Group 3 based on a host factor.

	Group 1	Group 2	Group 3	Total
drug j	$n_{ij}^{(1)}$	$n_{ij}^{(2)}$	$n_{ij}^{(3)}$	n_{ij}
no drug j	$n_{i.}^{(1)} - n_{ij}^{(1)}$	$n_{i.}^{(2)} - n_{ij}^{(2)}$	$n_{i.}^{(3)} - n_{ij}^{(3)}$	$n_{i.} - n_{ij}$
Total	$n_{i.}^{(1)}$	$n_{i.}^{(2)}$	$n_{i.}^{(3)}$	$n_{i.}$

An example for host factors with more than two levels

It is straightforward to extend the likelihood ratio test to situations where the host factor has more than two levels. The key is to use the multinomial distribution instead of the binomial distribution. Here, we illustrate it with an example for which the host factor has three levels. Table A.5 is similar to Table 2 except that there are now three groups for the host factor. Again, we denote $n_{ij}^{(s)}$ to be the number of reports for the ith AE and the jth drug regarding patients of the host factor group s ($s = 1, 2, 3$ for Groups 1, 2, and 3 respectively). Assume that $n_{ij}^{(s)}$ follows a Poisson($\mu_{ij}^{(s)}$) distribution with $\mu_{ij}^{(s)} = \lambda_{ij}^{(s)} E_{ij}^{(s)}$ and $E_{ij}^{(s)} = n_{i.}^{(s)} n_{ij}/n_{i.}$. The global hypothesis is thus $H_0 : \lambda_{ij}^{(1)} = \lambda_{ij}^{(2)} = \lambda_{ij}^{(3)} = 1$ for all j, versus $H_a : \lambda_{ij}^{(s)} \neq 1$ for at least one j and s. We can show that under the null hypothesis and conditioning on row and column totals,

$$n_{ij}^{(s)} | n_{ij}, n_{i.}^{(s)}, n_{i.} \sim \text{multinomial}\Big(n_{ij}, \frac{n_{i.}^{(s)}}{n_{i.}}\Big).$$

Note $n_{ij}^{(1)} + n_{ij}^{(2)} + n_{ij}^{(3)} = n_{ij}$. Correspondingly, we can derive the log likelihood ratio statistic as

$$\begin{aligned} LR_{ij} &= -\log\left(\frac{\max_{H_0} L(\lambda)}{\max_{H_a} L(\lambda)}\right) \\ &= -n_{ij}\log\left(\frac{n_{ij}}{n_{i.}}\right) + n_{ij}^{(1)}\log\left(\frac{n_{ij}^{(1)}}{n_{i.}^{(1)}}\right) + n_{ij}^{(2)}\log\left(\frac{n_{ij}^{(2)}}{n_{i.}^{(2)}}\right) + n_{ij}^{(3)}\log\left(\frac{n_{ij}^{(3)}}{n_{i.}^{(3)}}\right). \end{aligned}$$

The p-value for the LR statistic can be computed using Monte Carlo simulations as described previously.
