# Supplementary material for: Statistical methods for exploring spontaneous adverse event reporting databases for drug-host factor interactions
Source: BMC Med Res Methodol. 2023 Mar 27;23:71. doi: 10.1186/s12874-023-01885-w (PMC10041785; doi:10.1186/s12874-023-01885-w)
Supplement: Supplementary file 2 — Additional file 2. Mathematical derivations. [file 12874_2023_1885_MOESM2_ESM.pdf]

Mathematical Derivations

Mathematical derivations for some results given in the Methods section.

1 Normal approximation test as the test for difference in proportions

Another way to derive the normal approximation test is to consider the difference in proportions. Under the null hypothesis, the proportion of the two subgroups among those taking the drug or not taking the drug should be equal, that is,

$$H_0 : E\left[\frac{n_{ij}^{(1)}}{n_{ij}}\right] = E\left[\frac{n_{i\cdot}^{(1)} - n_{ij}^{(1)}}{n_{i\cdot} - n_{ij}}\right].$$

This formulation resembles the commonly used difference in proportions test, and therefore motivates performing inference based on the difference between $n_{ij}^{(1)}/n_{ij}$ and $[n_{i\cdot}^{(1)} - n_{ij}^{(1)}]/[n_{i\cdot} - n_{ij}]$.

Construct a test statistic for the difference in proportions as the following:

$$\begin{aligned}
\frac{n_{ij}^{(1)}}{n_{ij}} - \frac{n_{i\cdot}^{(1)} - n_{ij}^{(1)}}{n_{i\cdot} - n_{ij}} &= \frac{n_{ij}^{(1)}}{n_{ij}} + \frac{n_{ij}^{(1)}}{n_{ij}} \cdot \frac{n_{ij}}{n_{i\cdot} - n_{ij}} - \frac{n_{i\cdot}^{(1)}}{n_{i\cdot} - n_{ij}} \\
&= \frac{n_{ij}^{(1)}}{n_{ij}}\left(1 + \frac{n_{ij}}{n_{i\cdot} - n_{ij}}\right) - \frac{n_{i\cdot}^{(1)}}{n_{i\cdot} - n_{ij}} \\
&= \frac{n_{ij}^{(1)}}{n_{ij}} \cdot \frac{n_{i\cdot}}{n_{i\cdot} - n_{ij}} - \frac{n_{i\cdot}^{(1)}}{n_{i\cdot}} \cdot \frac{n_{i\cdot}}{n_{i\cdot} - n_{ij}} \\
&= \frac{n_{i\cdot}}{n_{i\cdot} - n_{ij}}\left(\frac{n_{ij}^{(1)}}{n_{ij}} - \frac{n_{i\cdot}^{(1)}}{n_{i\cdot}}\right). \qquad (1)
\end{aligned}$$

As the proposed probability model conditions on the values of n_{ij}, $n_{i\cdot}^{(1)}$, and $n_{i\cdot}$, the only random component in the last expression would be the ratio $n_{ij}^{(1)}/n_{ij}$, and the values $\frac{n_{i\cdot}}{n_{i\cdot} - n_{ij}}$ and $\frac{n_{i\cdot}^{(1)}}{n_{i\cdot} - n_{ij}}$ are treated as constants. Since we have the normal approximation

$$\sqrt{n_{ij}}\left(\frac{n_{ij}^{(1)}}{n_{ij}} - \frac{n_{i\cdot}^{(1)}}{n_{i\cdot}}\right) \overset{approx.}{\sim} N\left(0, \frac{n_{i\cdot}^{(1)}}{n_{i\cdot}}\left(1 - \frac{n_{i\cdot}^{(1)}}{n_{i\cdot}}\right)\right),$$

this lets us to approximate a centered and scaled version of (1) with a normal distribution for large n_{ij}, as we can relate the expressions of (1) and (2) by a scalar factor:

$$\begin{aligned}
&\sqrt{n_{ij}}\left(\frac{n_{ij}^{(1)}}{n_{ij}} - \frac{n_{i\cdot}^{(1)} - n_{ij}^{(1)}}{n_{i\cdot} - n_{ij}}\right) \\
= \quad & \frac{n_{i\cdot}}{n_{i\cdot} - n_{ij}}\left[\sqrt{n_{ij}}\left(\frac{n_{ij}^{(1)}}{n_{ij}} - \frac{n_{i\cdot}^{(1)}}{n_{i\cdot}}\right)\right] \\
\overset{approx.}{\sim} \quad & N\left(0, \frac{n_{i\cdot}^{(1)}}{n_{i\cdot}}\left(1 - \frac{n_{i\cdot}^{(1)}}{n_{i\cdot}}\right)\left(\frac{n_{i\cdot}}{n_{i\cdot} - n_{ij}}\right)^2\right). \qquad (2)
\end{aligned}$$

When performing inference, this leads to the z-score value of

$$\begin{aligned}
&\sqrt{n_{ij}}\left(\frac{n_{ij}^{(1)}}{n_{ij}} - \frac{n_{i\cdot}^{(1)} - n_{ij}^{(1)}}{n_{i\cdot} - n_{ij}}\right)\left(\frac{n_{i\cdot}^{(1)}}{n_{i\cdot}}\left(1 - \frac{n_{i\cdot}^{(1)}}{n_{i\cdot}}\right)\left(\frac{n_{i\cdot}}{n_{i\cdot} - n_{ij}}\right)^2\right)^{-1/2} \\
= \quad & \sqrt{n_{ij}} \cdot \frac{n_{i\cdot}}{n_{i\cdot} - n_{ij}}\left(\frac{n_{ij}^{(1)}}{n_{ij}} - \frac{n_{i\cdot}^{(1)}}{n_{i\cdot}}\right) \Bigg/ \left(\frac{n_{i\cdot}}{n_{i\cdot} - n_{ij}}\sqrt{\frac{n_{i\cdot}^{(1)}}{n_{i\cdot}}\left(1 - \frac{n_{i\cdot}^{(1)}}{n_{i\cdot}}\right)}\right) \\
= \quad & \sqrt{n_{ij}}\left(\frac{n_{ij}^{(1)}}{n_{ij}} - \frac{n_{i\cdot}^{(1)}}{n_{i\cdot}}\right) \Bigg/ \sqrt{\frac{n_{i\cdot}^{(1)}}{n_{i\cdot}}\left(1 - \frac{n_{i\cdot}^{(1)}}{n_{i\cdot}}\right)}.
\end{aligned}$$

The final expression is exactly the z-score obtained with the normal approximation method.

2 Asymptotic properties for the proportional reporting ratio (PRR)

The log of the PRR can be re-written as

$$\begin{aligned}
\log PRR_{ij} &= \log\left(\frac{n_{ij}^{(1)}/n_{ij}}{(n_{i\cdot}^{(1)}-n_{ij}^{(1)})/(n_{i\cdot}-n_{ij})}\right) \\
&= \log\left(\frac{n_{ij}^{(1)}}{n_{ij}}\right) - \log\left(\frac{n_{i\cdot}^{(1)}-n_{ij}^{(1)}}{n_{i\cdot}-n_{ij}}\right) \\
&= \log\left(\frac{n_{ij}^{(1)}}{n_{ij}}\right) - \log\left(\frac{n_{i\cdot}^{(1)}-n_{ij}^{(1)}}{n_{ij}}\cdot\frac{n_{ij}}{n_{i\cdot}-n_{ij}}\right) \\
&= \log\left(\frac{n_{ij}^{(1)}}{n_{ij}}\right) - \log\left(\frac{n_{i\cdot}^{(1)}}{n_{ij}}-\frac{n_{ij}^{(1)}}{n_{ij}}\right) - \log\left(\frac{n_{ij}}{n_{i\cdot}-n_{ij}}\right). \qquad (3)
\end{aligned}$$

As the proposed probability model conditions on the values of n_{ij}, $n_{i\cdot}^{(1)}$, and $n_{i\cdot}$, the only random component in the last expression would be the ratio $n_{ij}^{(1)}/n_{ij}$, and all other terms in the expression can be treated as constants. This motivates the definition of the function f, where

$$f(x) := \log(x) - \log\left(\frac{n_{i\cdot}^{(1)}}{n_{ij}} - x\right) - \log\left(\frac{n_{ij}}{n_{i\cdot}-n_{ij}}\right).$$

Hence, we have $\log PRR_{ij} = f(n_{ij}^{(1)}/n_{ij})$. By taking derivative, it follows that

$$f'(x) = \frac{1}{x} + \frac{1}{\left(n_{i\cdot}^{(1)}/n_{ij}\right)-x} = \frac{n_{i\cdot}^{(1)}/n_{ij}}{x\left(\left(n_{i\cdot}^{(1)}/n_{ij}\right)-x\right)} = \frac{n_{i\cdot}^{(1)}}{x\left(n_{i\cdot}^{(1)}-n_{ij}x\right)}.$$

Letting $p_i = n_{i\cdot}^{(1)}/n_{i\cdot}$, one can also work backwards as in (3) to show that

$$f(p_i) = \log\left(\frac{p_i}{(n_{i\cdot}^{(1)}-n_{ij}p_i)/(n_{i\cdot}-n_{ij})}\right).$$

Working from the normal approximation

$$\sqrt{n_{ij}}\left(\frac{n_{ij}^{(1)}}{n_{ij}} - p_i\right) \overset{approx.}{\sim} N\left(0, p_i(1-p_i)\right), \qquad \text{where} \qquad p_i := \frac{n_{i\cdot}^{(1)}}{n_{i\cdot}},$$

we can use the delta method to obtain

$$\sqrt{n_{ij}}\left(\log PRR_{ij} - \log\left(\frac{p_i}{(n_{i\cdot}^{(1)}-n_{ij}p_i)/(n_{i\cdot}-n_{ij})}\right)\right) \overset{approx.}{\sim} N\left(0, p_i(1-p_i)\left(\frac{n_{i\cdot}^{(1)}}{p_i\left(n_{i\cdot}^{(1)}-n_{ij}p_i\right)}\right)^2\right)$$

for large values of n_{ij}.

3 Asymptotic properties of the reporting odds ratio (ROR)

We assume that $n_{i\cdot}^{(1)} > n_{ij}^{(1)}$ and $n_{i\cdot}^{(2)} > n_{ij}^{(2)}$. For real data examples, this is usually true; $n_{i\cdot}^{(s)}$ contains all counts from all drugs for AE i and subgroup s whereas $n_{ij}^{(s)}$ places a further upper limit to drug j. Only in the case where AE i and group s has reports from a single drug can we see cases where $n_{i\cdot}^{(s)} = n_{ij}^{(s)}$ for $s = 1$ or 2. But such scenarios were not encountered in our analysis, possibly due to our data cleaning. This does have implication for simulation studies,

for which one need careful selection of the simulation parameters n_{ij}, $n_{i\cdot}^{(s)}$, and $n_{i\cdot}$ for all i, j, s to avoid violating this assumption.

When $n_{i\cdot}^{(1)} > n_{ij}^{(1)}$ and $n_{i\cdot}^{(2)} > n_{ij}^{(2)}$ are satisfied, the log of the reporting odds ratio can be written as the following:

$$\begin{aligned}
\log ROR_{ij} &= \log\left(\frac{n_{ij}^{(1)}/n_{ij}^{(2)}}{\left(n_{i\cdot}^{(1)} - n_{ij}^{(1)}\right) / \left(n_{i\cdot}^{(2)} - n_{ij}^{(2)}\right)}\right) \\
&= \log\left(\frac{n_{ij}^{(1)}}{n_{i\cdot}^{(1)} - n_{ij}^{(1)}}\right) - \log\left(\frac{n_{ij}^{(2)}}{n_{i\cdot}^{(2)} - n_{ij}^{(2)}}\right) \\
&= \log\left(\frac{n_{ij}^{(1)}/n_{ij}}{n_{i\cdot}^{(1)}/n_{ij} - n_{ij}^{(1)}/n_{ij}}\right) - \log\left(\frac{n_{ij} - n_{ij}^{(1)}}{n_{i\cdot}^{(2)} - (n_{ij} - n_{ij}^{(1)})}\right) \\
&= \log\left(\frac{n_{ij}^{(1)}/n_{ij}}{n_{i\cdot}^{(1)}/n_{ij} - n_{ij}^{(1)}/n_{ij}}\right) - \log\left(\frac{1 - n_{ij}^{(1)}/n_{ij}}{n_{i\cdot}^{(2)}/n_{ij} - (1 - n_{ij}^{(1)}/n_{ij})}\right) \\
&= \log\left(\frac{n_{ij}^{(1)}}{n_{ij}}\right) - \log\left(\frac{n_{i\cdot}^{(1)}}{n_{ij}} - \frac{n_{ij}^{(1)}}{n_{ij}}\right) - \log\left(1 - \frac{n_{ij}^{(1)}}{n_{ij}}\right) + \log\left(\frac{n_{i\cdot}^{(2)}}{n_{ij}} - 1 + \frac{n_{ij}^{(1)}}{n_{ij}}\right) \\
&= f\left(\frac{n_{ij}^{(1)}}{n_{ij}}\right), \qquad (4)
\end{aligned}$$

where

$$f(x) := \log(x) - \log\left(\frac{n_{i\cdot}^{(1)}}{n_{ij}} - x\right) - \log(1-x) + \log\left(\frac{n_{i\cdot}^{(2)}}{n_{ij}} - 1 + x\right).$$

With this definition, it follows that

$$\begin{aligned}
f'(x) &= \frac{1}{x} + \frac{1}{\frac{n_{i\cdot}^{(1)}}{n_{ij}} - x} + \frac{1}{1-x} + \frac{1}{\frac{n_{i\cdot}^{(2)}}{n_{ij}} - 1 + x} \\
&= \frac{n_{i\cdot}^{(1)}/n_{ij}}{x\left(\frac{n_{i\cdot}^{(1)}}{n_{ij}} - x\right)} + \frac{n_{i\cdot}^{(2)}/n_{ij}}{(1-x)\left(\frac{n_{i\cdot}^{(2)}}{n_{ij}} - 1 + x\right)}.
\end{aligned}$$

Letting $p_i := n_{i\cdot}^{(1)}/n_{i\cdot}$, again, we can also work backwards as in (4) to show that

$$\begin{aligned}
f(p_i) &= \log\left(\frac{p_i}{\frac{n_{i\cdot}^{(1)}}{n_{ij}} - p_i}\right) - \log\left(\frac{1-p_i}{\frac{n_{i\cdot}^{(2)}}{n_{ij}} - (1-p_i)}\right) \\
&= \log\left(\frac{p_i/(1-p_i)}{\left(\frac{n_{i\cdot}^{(1)}}{n_{ij}} - p_i\right) / \left(\frac{n_{i\cdot}^{(2)}}{n_{ij}} - 1 + p_i\right)}\right).
\end{aligned}$$

Using the delta method as in Section 2, it follows that for large n_{ij}, we have

$$\begin{aligned}
&\sqrt{n_{ij}}\left(\log ROR_{ij} - \log\left(\frac{p_i/(1-p_i)}{\left(\frac{n_{i\cdot}^{(1)}}{n_{ij}} - p_i\right) / \left(\frac{n_{i\cdot}^{(2)}}{n_{ij}} - 1 + p_i\right)}\right)\right) \\
&= \sqrt{n_{ij}}\left(f\left(\frac{n_{ij}^{(1)}}{n_{ij}}\right) - f(p_i)\right) \\
&\overset{approx.}{\sim} N(0, p_i(1-p_i)(f'(p_i))^2) \\
&= N\left(0, p_i(1-p_i)\left(\frac{n_{i\cdot}^{(1)}/n_{ij}}{p_i\left(\frac{n_{i\cdot}^{(1)}}{n_{ij}} - p_i\right)} + \frac{n_{i\cdot}^{(2)}/n_{ij}}{(1-p_i)\left(\frac{n_{i\cdot}^{(2)}}{n_{ij}} - 1 + p_i\right)}\right)^2\right).
\end{aligned}$$
